# The simulated early learning of cervical spine manipulation technique utilising mannequins

**DOI:** 10.1186/s12998-015-0067-6

**Published:** 2015-08-03

**Authors:** Peter D Chapman, Norman J Stomski, Barrett Losco, Bruce F Walker

**Affiliations:** 6 Claredon Court, Alexander Heights, 6064 WA Australia; School of Health Professions, Murdoch University, 90 South St, Murdoch, 6150 WA Australia; Discipline of Chiropractic, School of Health Professions, Murdoch University, 90 South St, Murdoch, 6150 WA Australia

**Keywords:** Chiropractic, Education, Neck manipulation, Randomised trial, Mannequin, Simulated learning

## Abstract

**Background:**

Trivial pain or minor soreness commonly follows neck manipulation and has been estimated at one in three treatments. In addition, rare catastrophic events can occur. Some of these incidents have been ascribed to poor technique where the neck is rotated too far. The aims of this study were to design an instrument to measure competency of neck manipulation in beginning students when using a simulation mannequin, and then examine the suitability of using a simulation mannequin to teach the early psychomotor skills for neck chiropractic manipulative therapy.

**Methods:**

We developed an initial set of questionnaire items and then used an expert panel to assess an instrument for neck manipulation competency among chiropractic students. The study sample comprised all 41 fourth year 2014 chiropractic students at Murdoch University. Students were randomly allocated into either a usual learning or mannequin group. All participants crossed over to undertake the alternative learning method after four weeks. A chi-square test was used to examine differences between groups in the proportion of students achieving an overall pass mark at baseline, four weeks, and eight weeks.

**Results:**

This study was conducted between January and March 2014. We successfully developed an instrument of measurement to assess neck manipulation competency in chiropractic students. We then randomised 41 participants to first undertake either “usual learning” (n = 19) or “mannequin learning” (n = 22) for early neck manipulation training. There were no significant differences between groups in the overall pass rate at baseline (χ^2^ = 0.10, *p* = 0.75), four weeks (χ^2^ = 0.40, *p* = 0.53), and eight weeks (χ^2^ = 0.07, *p* = 0.79).

**Conclusions:**

This study demonstrates that the use of a mannequin does not affect the manipulation competency grades of early learning students at short term follow up. Our findings have potentially important safety implications as the results indicate that students could initially gain competence in neck manipulation by using mannequins before proceeding to perform neck manipulation on each other.

## Background

Neck manipulation is a commonly used manual therapy for pain and stiffness and is likely to have been practised for centuries [1]. In more modern times it has become even more popular as a technique used by chiropractors and others; however it has been a source of controversy as to its risk/benefit ratio [2, 3]. Complications associated with neck manipulation have been estimated at a rate of one in three treatments but these adverse events are usually negligible causing minor soreness [4]. However, rare catastrophic events can occur. Over the past 30 years there has been a growing awareness of complications arising from the use of neck manipulation, in particular, stroke has become a prominent and worrying concern for practitioners of manual therapy and patients [5–10]. This adverse event may result in permanent impairment or more rarely death. Estimates of vertebral artery stroke (VAS) incidence following neck manipulation are likely to be between 1 in 400,000 cervical manipulations [11] and 1 in 100,000 patients receiving cervical spinal manipulative therapy (SMT) [5].

Some of these serious incidents have been ascribed to poor manipulative technique where the neck is excessively rotated. In a University setting where inexperienced students are learning manipulation it may be logical to speculate that these un-skilled operators may deliver a technique that contributes towards a greater complication risk.

The current method of teaching students the psychomotor skills of manipulation is to have students practice on each other. This is problematic for several reasons; a) the necessary skills (speed and accuracy) are not yet present in the student; b) a force that is not fully controlled is being put through the joints of fellow students and c) joints that do not need manipulation are being manipulated. In total this may lead to over stretching of the joint tissues with resulting soreness. To become proficient at the skill of neck manipulation a great amount of practice is needed and to obtain mastery a student has to practice many repetitive movements [12]. This usually means that over the course of an undergraduate chiropractic program the students may perform hundreds of manipulations on each other, with consequent increases in the risk of adverse reactions of varying severities. We found no evidence that stroke has occurred during neck manipulation training however it is intuitive that adverse events are more likely during this first phase of skills training. For example an epidemiological survey of chiropractic students enrolled in the chiropractic program offered at Parker College of Chiropractic in Dallas, Texas, USA revealed that 25 % (143/572) of respondents reported an injury as a result of manipulation being performed on them by other students during their studies [13]. These results are lower than those reported by an earlier study where 55 % of study participants experienced an injury as a result of learning to perform spinal manipulation [14].

Ndetan et al. (2009) reported that the most frequently injured region of the body as a result of students receiving spinal manipulation as part of their training was the neck and shoulder region [13]. However, Macanuel et al. (2005) differ slightly and suggest that this region is the second most commonly injured region, with the lumbopelvic region being the most commonly injured region, amongst chiropractic students when learning to perform spinal manipulative therapy [14].

It has been suggested that chiropractic students may be exposed to injuries to the cervical spine as a result of receiving “amateurish adjustments” or manipulations that deliver a substantial rotatory effect upon the cervical spine from other students [13].

It is therefore important in the early phases of training to search for innovative methods such as simulation to assist chiropractic students achieve a level of mastery of the manipulation techniques but with a low risk of adverse events. To this end we hypothesise that it may be useful to have students reach a level of proficiency by practicing on a mannequin with a flexible neck that approximates the resistance of a human cervical spine. To date very little research has been published on cervical manipulation mannequins and whether they assist the student with skill mastery or the effect upon student safety.

The teaching of spinal manipulative techniques at Murdoch University is consistent with that described by Harvey et al. [15]. Students are first introduced to the theoretical aspects of spinal manipulation by attending didactic lectures in areas such as anatomy, biomechanics and spinal manipulative technique, they then combine this knowledge with the subjective assessment of observing an instructor/expert perform spinal manipulative techniques. The students then progress to copy the demonstrated movements, which usually involves one student acting as a simulated patient while another student attempts to replicate the instructor’s execution of the technique. This includes correct hand positions, patient pre-positioning, as well as the direction and magnitude of force required to perform the manipulative technique. Instructors then provide qualitative verbal feedback.

The aims of this study were first to design an instrument to measure competency of neck manipulation technique, and then to use this instrument to measure the suitability of using a simulation mannequin to teach the necessary psychomotor skills for chiropractic manipulative therapy of the neck.

## Methods

### Questionnaire development

We developed an initial set of questionnaire items in consultation with Murdoch staff members who taught cervical manipulation technique. An expert panel was then formed, who used a Content Validity Index (CVI) to ensure that the questionnaire was relevant to assess neck manipulation competency among chiropractic students [16, 17]. The expert panel consisted of seven chiropractors who teach neck manipulation at several Australian universities and one international university. The composition and size of this expert panel was congruent with guidelines that propose that the panel members’ professional backgrounds reflect that of the target population, and that the ideal number is between six to twelve members [16, 17]. Every panel member evaluated each item using four categories: not relevant, unable to assess relevance without major revision, relevant but needs minor alteration, and very relevant. A value of one was assigned to the “very relevant” and “relevant but needs minor alteration” categories, whereas a value of zero was assigned to the other categories. The I-CVI for each item was derived by summing the values for each rater and then dividing by the number of raters. Items were retained if the CVI exceeded 0.79 [16, 17]. After the initial expert panel evaluation and feedback, the pilot version of the questionnaire was expanded considerably in the domains of rating expertise. In the second round of evaluation, the I-CVI for individual items ranged from 0.86-1.0 and all items were retained. The S-CVI, which is the proportion of items rated as either “relevant but needs minor alteration” or “very relevant” by all raters, was 0.94.

The final questionnaire comprised five assessment criteria, of which three contained several sub-criteria. The five assessment criteria were:

1. Patient and practitioner positioning
  Is the patient positioned correctly?
  Is the headrest in the correct position?
  Is the practitioner positioned correctly?
2. Joint pre-tension
  Has the joint been placed into the pre-manipulative tension position correctly?
3. Contact points
  Is the contact point on the patient correct?
  Has the correct side of the patient been contacted?
  Is the contact on the practitioner’s hand correct?
  Is the practitioner’s indifferent/stabilising hand correctly positioned?
4. Vector of correction/line of drive
  Is the vector of correction correctly aligned with the presentation of the facet joints at the level manipulated?
5. Procedure
  Is the amplitude of the thrust applied sufficient to address the fixation?
  Is the speed of the thrust applied sufficient to address the fixation?
  Was the demonstrated manipulation/adjustment executed safely?

### Sample

All students enrolled in the fourth of the five year chiropractic program at Murdoch University during 2014 (N = 41) were invited to participate. Paper copy information notices were distributed in the first lecture of 2014, and a non-teaching staff member delivered an information session and provided an opportunity for students to raise questions about the study to inform the consent process. Students were informed that participation was entirely voluntary, and electing to participate or not participate, would not affect their relationship with University staff in any way. The Murdoch University Human Research Ethics Committee approval number was 2013/200. All 41 students initially invited to participate provided consent to have their results included in the final analysis.

### Randomisation and blinding procedure

A staff member, not involved with group allocation, used a random number generator to generate a randomisation list. The group assignment was placed in sequentially numbered, opaque, sealed envelopes. After obtaining informed consent, staff not assessing neck manipulation competency opened the envelope and allocated students to one of two groups: usual learning or mannequin learning. Staff assessing neck manipulation competency, and undertaking data entry and analysis were blinded to group allocation.

Due to the nature of the study it was not possible to blind students to their group allocation, however at all times the assessor remained unaware of the group randomisation. Students are aware that under Australian Law, unless they are a registered practitioner with the Australian Health Professions Regulatory Authority, performing cervical spine manipulation outside of a recognised training program is prohibited [18]. Thus it is unlikely that students practised cervical spine manipulation outside of their usual teaching times.

### The mannequin

**Fig. 1:**
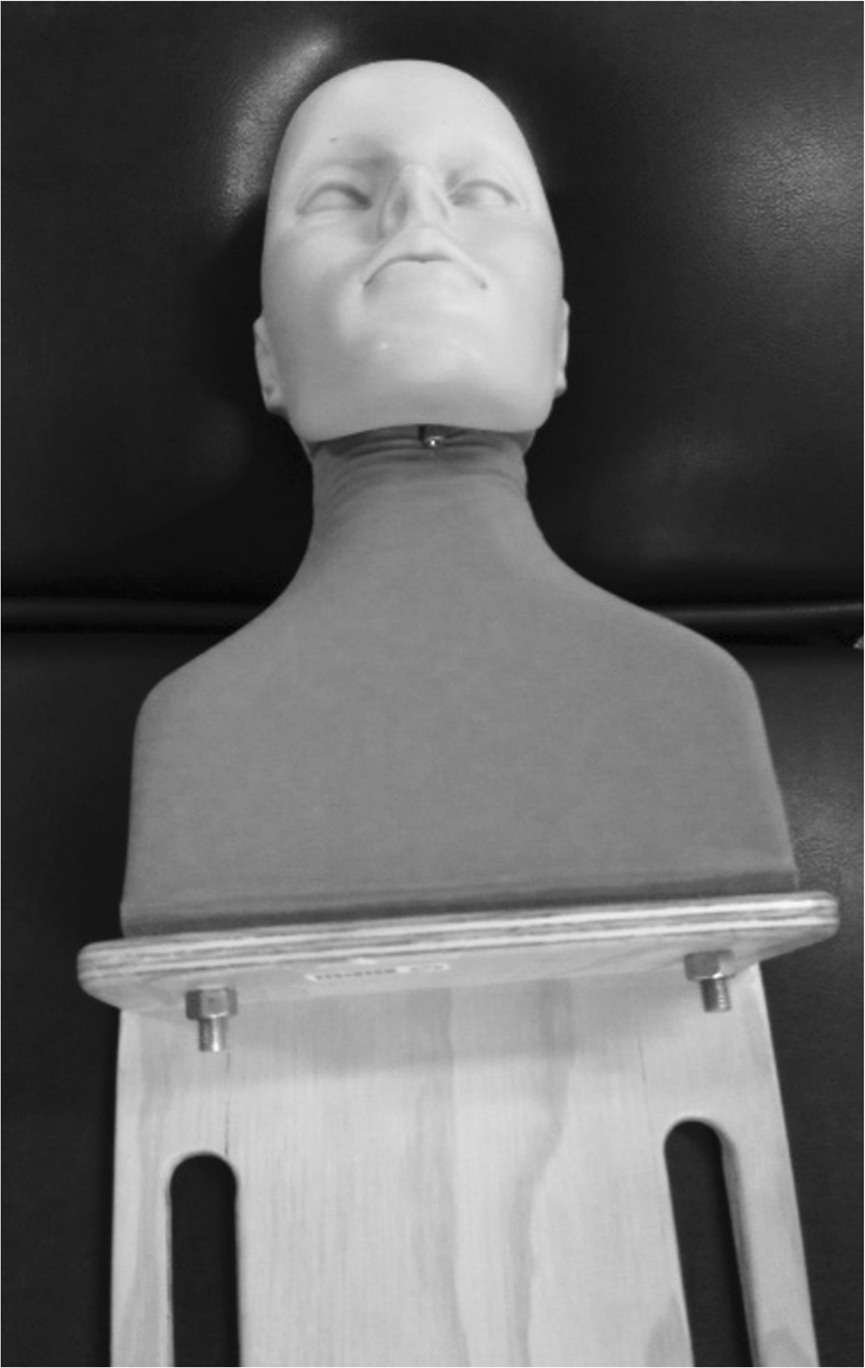
The mannequin

**Fig. 2:**
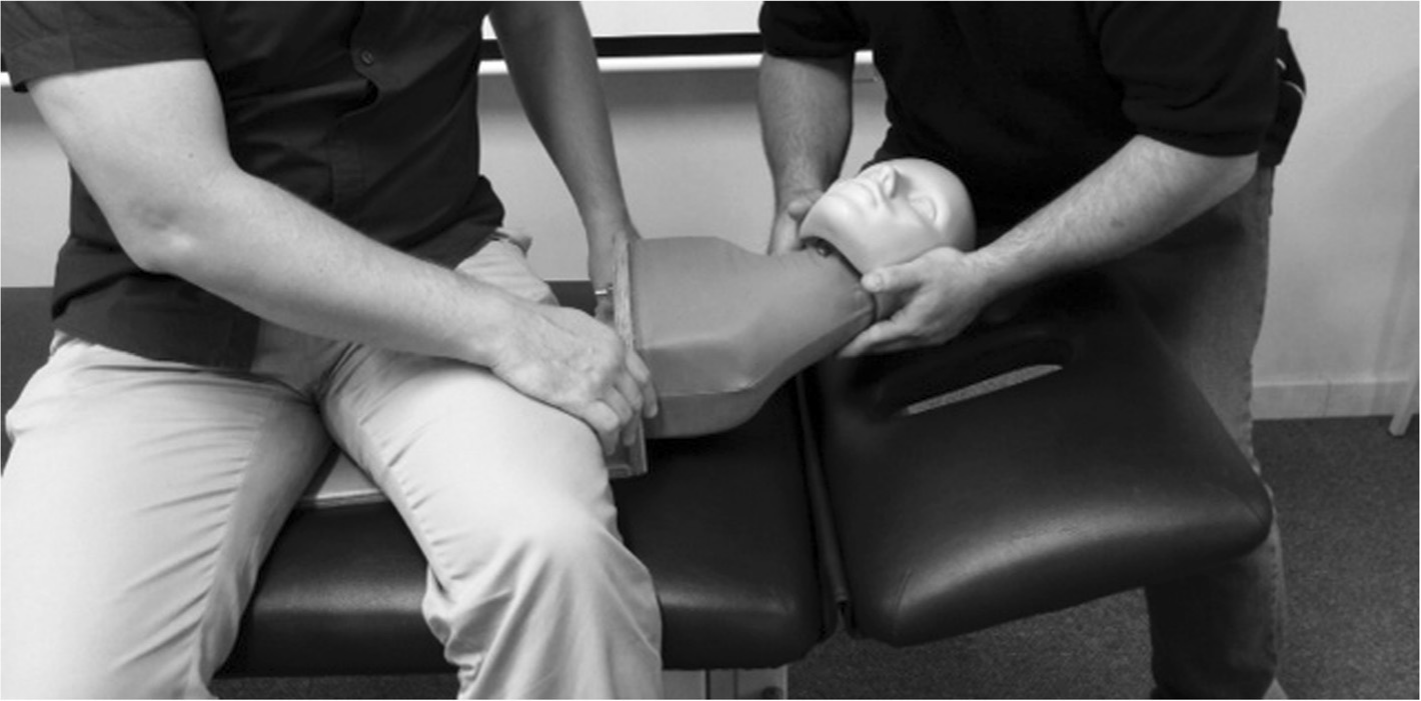
Technique demonstration on the mannequin

The cervical mannequin is known as “Flexi-man” and is shown in Figs. 1 & 2. Fleximan has been developed and manufactured by Dr. Timothy Young, Chiropractor [19]. The mannequin consists of a flexible imitation of shoulders, neck and head made of a pliant “rubberised” material. The weight of the mannequin is 4.8 kgs with the specific head weight unknown, however a proxy head weight was 3.2 kgs. This proxy weight was established by laying the mannequin prone with the head recumbent on weight scales. The mannequin was designed to allow students to set up, place contacts and deliver a thrust in a line of drive of their choosing. It does not however allow for pre-manipulative tension. The mannequin is a stylised human facsimile and is not designed to mimic a human specimen. It does not have the variability of human subjects receiving manipulation, for example height, weight, and tissue compliance. The makers state that the mannequin is best used in the introductory phases of training [20].

### Educational interventions

**Fig. 3:**
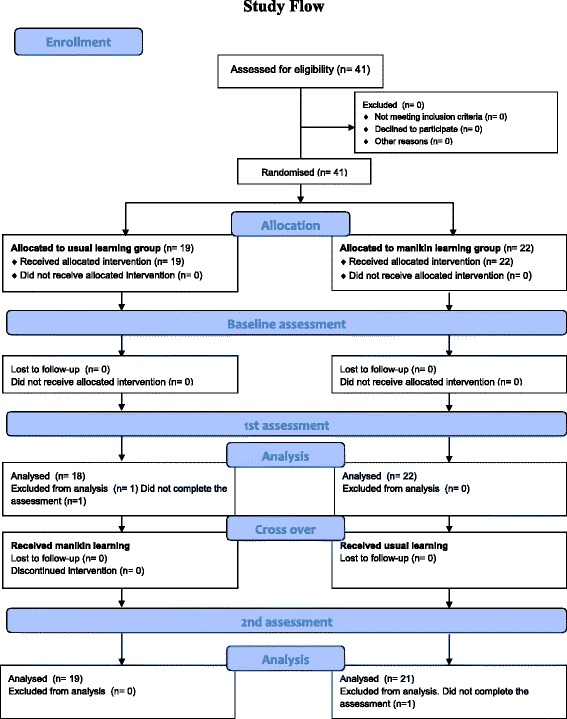
Flow chart of study

The usual learning group practised neck manipulation techniques on each other under supervision consistent with the description provided in the introduction to this article. This learning approach has been used since the inception of the Murdoch University chiropractic program in 2002. The mannequin learning group practiced the neck manipulation techniques on a mannequin with a flexible neck, once again under supervision. The mannequin learning approach was a novel method not previously used at Murdoch University. Each group received three, two hour weekly training sessions in the performance of a commonly used cervical spine manipulation technique referred to as the index pillar push [21]. During the weekly training sessions and to ensure that the assessor remained blinded to group allocation students were supervised by academics not involved in the assessment of the students. The supervising academics provided each student with regular personalised feedback relating to their performance of the required technique during the weekly training sessions. After the third weekly training session, all members of each group crossed over and undertook three additional training sessions using the alternate method. The flow of the study is displayed in Fig. 3.

All fourth year students who participated in the study had already received training in neck manipulation in the third year of the program but there is a long summer break between third and fourth year so skills usually diminish. On the first week back at University, a staff member experienced in manipulation assessment who was blinded to group randomisation, assessed the student’s performance of the index pillar push procedure at baseline on the mannequin (immediately before the first training session) and then again at four weeks, and eight weeks, by using the validated instrument of measurement.

Assessing student performance of the index pillar push technique involved the assessor requesting the student to perform the technique on both the left and the right hand side of the mannequin. Each student was allowed to perform the technique once on both sides, while the assessor graded their performance.

The maximum total score on the validated questionnaire is 100 %. In order to pass the assessment a student must achieve a minimum score of 69 % overall.

Each of the five identified assessment criteria are considered critical to the successful performance of the required technique. If a student fails to perform one of these five assessment criteria adequately the student is awarded an overall score of 69 %. Should the student fail to perform two of the five assessment criteria adequately they are awarded a score of 49 %. Less than satisfactory performance of three criteria results in a score of 29 %. Inadequate performance of four assessment criteria results in a score of 19 % being awarded to the student. If all five assessment criteria are unsatisfactorily performed or the student is unable to perform the required technique, the student is awarded a score of zero.

### Statistical analysis

All data were entered manually into SPSS v.21, cleaned and checked for implausibilities. A chi-square test was used to examine differences between groups for the proportion of students achieving an overall pass mark at baseline, four weeks, and eight weeks.

## Results

This study was conducted between January and March 2014. We randomised 41 participants to first undertake either usual learning (n = 19) or mannequin (n = 22). The proportion of female participants was 36.8 % in the usual learning group, and 45.4 % in the mannequin group. The mean age was 23.3 (SD 4.7) years in the usual learning group, and 22.8 (SD 3.4) years in the mannequin group. All participants crossed over to undertake the alternative learning method after four weeks.

There were no significant differences between groups in the overall pass rate at baseline (χ^2^ = 0.10, p = 0.75), four weeks (χ^2^ = 0.40, p = 0.53), or at eight weeks (χ^2^ = 0.07, p = 0.79). The proportion of participants achieving an overall pass in each group at each time point is displayed in Table 1. There were no reported adverse events in the students’ group from the therapy administered.

**Table 1:** Overall pass rate for cervical manipulation

	Number of participants achieving overall pass at baseline	Number of participants achieving overall pass at four weeks	Number of participants achieving overall pass at eight weeks
Participants first undertaking usual learning (n = 19)	22.2 % (4/18)	44.4 % (8/18)	84.2 % (16/19)
Participants first undertaking mannequin training (n = 22)	18.2 % (4/22)	54.5 % (12/22)	81.0 % (17/21)

## Discussion

Our findings show that mannequins can be used to teach cervical neck manipulation technique without affecting the grades for neck manipulation competency of chiropractic students. This result was consistent with a previous study examining the use of mannequins in teaching cervical manipulation [22]. These findings have important implications for the delivery of chiropractic curricula because the use of mannequins reduces the number of manipulations received by fellow students, which thereby improves the safety of chiropractic training.

In a previous study, differences in learning outcomes were examined by comparing students who undertook either practitioner-positioning training or complete practice training [15]. The practitioner-positioning approach involved students learning the components of spinal manipulation without ever delivering the thrust component. The complete practice approach incorporated the thrust component under instructor supervision and with proprioceptive feedback. This study was administered soon after students’ commenced training in the first year of a Master’s degree program at Macquarie University in Sydney, New South Wales, Australia which is the equivalent to the 4th year of Murdoch University’s program. The study reported that the complete practice approach resulted in significantly higher peak force, and significantly lower time to peak force, both of which are thought to demonstrate higher proficiency in performing spinal manipulation [15]. So, although including the thrust component early in training may enhance the acquisition of manipulation competency, it could also potentially increase the likelihood of students experiencing adverse events. Therefore, by introducing the mannequin into the early stages of students’ training, students could experience enhanced learning outcomes without any risk of decreased competency or adverse events.

Several studies have suggested that quantitative assessment of spinal manipulation is superior to qualitative assessment for the acquisition of psychomotor skills [23–25]. Traditionally, students were provided qualitative spinal manipulation feedback by instructors who evaluated parameters such as thrust vector, preload force, amplitude, velocity, and body position. Indeed, these parameters comprised the assessment criteria we derived for the instrument used in this study. Qualitative assessment may be prone to feedback inconsistency due to instructor experience, differing opinions between instructors, and observational oversights, which all may impede the acquisition of psychomotor skills. However, these inconsistencies can be addressed by the use of quantitative feedback devices that measure peak force or force time histories [25–27]. Quantitative feedback has an immediate positive impact upon all parameters relating to the performance of lumbar spine manipulation including subjective patient ratings of task performance [28], and has been shown to be superior to qualitative feedback [29]. Timely quantitative feedback allows the student to make rapid changes to their performance of the required technique [28]. Given this, we recommend that as a next step further research examine how apparatus capable of producing quantitative feedback could be most effectively incorporated with the use of cervical mannequins.

A strength of this study was the use of an expert panel to evaluate the content validity of the neck manipulation competency questionnaire. This resulted in the development of a questionnaire that is appropriate to assess the skills of chiropractic students learning neck manipulation. However, we did not assess the test-retest reliability of the questionnaire, and its reliability should be further determined in other studies. Another limitation of this study was not evaluating intra-rater examiner reliability, so the extent to which this may have influenced the findings remains unclear. Finally, we used a convenience sample so the study may have been underpowered however the results for a reasonable class size allowed us to conclude equivalence between the methods of teaching.

## Conclusions

This study consolidates previous research concerning the use of mannequins to teach neck manipulation skills and confirms that the use of mannequins does not affect the manipulation competency grades of students. Our findings have potentially important safety implications as the results indicate that students could initially gain competence in neck spinal manipulation by using mannequins before proceeding to perform neck manipulation on each other, which is likely to lessen the risk of students experiencing adverse events.
